# eHealth-Based Interventions for Older Patients with Prostate Cancer: A Quick Review of the Literature

**DOI:** 10.1089/tmr.2021.0048

**Published:** 2022-03-17

**Authors:** Luiz Sinésio Silva Neto, Fellipe Camargo Ferreira Dias, Neila Barbosa Osório, Carmem Lucia Artioli Rolim

**Affiliations:** ^1^ Faculty of Medicine, Federal University of Tocantins, Palmas, Brazil.; ^2^ University of Maturity, Federal University of Tocantins, Palmas, Brazil.; ^3^ Faculty of Education, Federal University of Tocantins, Palmas, Brazil.

**Keywords:** prostate cancer, internet-based intervention, aged

## Abstract

**Background:**

The prevalence of prostate cancer (PC) is higher in older adults. Due to early diagnosis and treatment, there is an increase in the survival rate of these patients. The survival of patients with PC imposes the need for specific and effective care strategies.

**Objective:**

To identify and analyze eHealth intervention programs for older adults with PC.

**Methods:**

A quick review of evidence from the current literature was employed to address the objective of the study. The recommendations of the Cochrane Rapid Reviews Methods Group were used. The PubMed, Embase, Capes Journals, and Lilacs-BVS databases were searched, covering studies published from January 2010 to July 2021. The articles selected were classified considering the modalities and type of eHealth strategies.

**Results:**

A total of 10 articles were included in this review. Two types of modalities were identified and classified: the intervention that used the web-based platform (WBP) was the most used in the studies (*n* = 7), followed by the interactive smartphone application (ISA) (*n* = 3) and mixed (WBP + ISA) (*n* = 1). As for the classification, mixed interventions were the most used (*n* = 4), followed by self-monitoring (*n* = 3), educational (*n* = 2), and behavioral counseling (*n* = 1). The clustering of articles generated three groups for the presentation of results and discussion, being eHealth interventions: integrated care, detection of symptoms, and quality of life in older patients with PC, psychological eHealth interventions in older adults with PC, and physical activity eHealth interventions in older adults with PC.

**Conclusion:**

eHealth interventions for patients with PC are relatively new but promising in the support of current care options.

## Background

Prostate cancer (PC) is one of the most common cancers diagnosed among men worldwide. The number of patients with PC will increase significantly due to the aging of the population.^1^ Older adults represent the age group most affected by this type of cancer—about 89.80% of all cases are in patients aged 60+ years, with the highest prevalence between 70 and 79 years.^2^ Improvements in early detection and treatment have increased cancer patient survival rates, with 84% of patients living for 10 years or more.^3^ This situation of a higher number of cases and longer survival rates of patients with PC imposes a growing demand for specialized services. A previous study demonstrated the continuous needs during and after treatment of PC patients.^4^

Older men may have more complications related to PC due to age-related comorbidities, such as vascular diseases, other types of cancer, and infections.^5^ In addition, another study showed that older adults often suffer from a decline in their basic skills, such as cognitive (e.g., decreased working memory) and sensory (e.g., decreased visual acuity) impairments, which makes their online experience different from adults younger than 60 years.^6^ Therefore, changes in the organization of services are necessary to ensure that these needs and others that impact health-related quality of life (HRQOL) are effectively met.

The use of the internet for health information and activities is growing rapidly, especially among older adults. In addition, web-based interventions and smartphone applications (eHealth) are used by some health professionals and researchers, being considered increasingly important tools.^7–9^ Therefore, eHealth interventions to better address the unmet needs of PC patients and their families is a growing field of research. Internet-based tools can help improve access to care, while allowing low-cost high-quality personalized care, greater access to a multidisciplinary team of health professionals (without needing to leave the household), convenience of participation anytime of the day, enabling patients to have access to care outside the normal business hours, and ability to provide not only high-quality content but also highly personalized content.

This is complemented by opportunities to interact with other people and tools designed to support self-management and decision-making.^10–12^ However, there is a concern that the internet may be perpetuating disparities in access to health services, keeping certain segments of the population, such as older adults, on the sidelines.^13^ In this sense, Penedo et al.^13^ highlighted that this digital gap can have important implications for people's access to information and health care, especially as a growing number of health care services have shifted to eHealth systems. Therefore, ensuring that appropriate and useful eHealth information about breast cancer is available to audiences at this stage of life is important, given these considerations.

Although eHealth intervention studies to improve the health of older patients with PC are increasing, it is still an underexplored field. Therefore, the objective of this review was to identify and analyze eHealth intervention programs for older adults with PC.

## Methodology

### Research question

What are the modalities and categories used in eHealth interventions for older adults with PC?

### Study design

This study is a quick review, following the recommendations proposed by the Cochrane Rapid Reviews Methods Group.^14^ The literature search was carried out on June 4, 2021. The search strategy was developed iteratively from the PubMed database and later adapted to the others. The final search strategy included four keyword groups with a combination of Medical Subject Headings (MeSH) and text keywords. We only included articles in the English language, related to human participants.

#### Source selection

The sources were available on the internet, in scientific databases in the area. Studies available in other media could also be selected, as long as they met the requirements of a quick review.

#### Keywords

Our search strategy was: (1) identification of keywords, (2) refinement of the selection of studies found, (3) reading the abstracts to select the studies corresponding to the inclusion criteria, and (4) reading texts in full as a final step. We searched the databases using the following keyword combinations: eHealth technology; Prostate cancer; Intervention AND Internet-Based Intervention; Older Adults OR Elderly or Aged.

**Table:** 

Search strings
eHealth technology AND Prostate cancer (tittle) AND Intervention AND Older Adults OR Elderly or Aged eHealth technology AND Prostate cancer (title) AND Intervention AND Older Adults AND Quality of life (eHealth technology) AND (ti:(prostate cancer)) (eHealth technology) AND (ti:(prostate cancer)) AND (intervention) (((eHealth technology) AND (prostate cancer CT)) AND (intervention)) AND (older adults)

#### Source listing

The criteria for choosing the indexers were chosen for being available on the internet and with free access. To systematize the information on the systematic review process, the computer software START^®^ version 2.03 was used.^15^ The purpose of this program was to assist in the Systematic Review process in all its stages: planning, execution, and analysis of results. Here are the sources used in the review: PubMed (https://pubmed.ncbi.nlm.nih.gov), Embase (https://www.embase.com), Capes Journals (http://novo.periodicos.capes.gov.br), and Lilacs-BVS (https://bvsalud.org). In Table 1 is the description of articles by databases.

**Table 1.** Description of Articles by Databases

Database	*N* (articles)	*N* (%)
PubMed	13	28
Embase	6	13
Portal Periódicos da Capes (Capes Journals)	17	36
Lilacs-BVS	11	23
Total	47	100

#### Inclusion and exclusion criteria

##### Inclusion criteria

(a) Studies published and fully available in scientific databases.
(b) Recent studies published from 2010 onward.
(c) Studies that exclusively address patients with PC will be included.

##### Exclusion criteria

(a) Studies that assess interventions in patients with PC, without using eHealth strategies.
(b) Studies published as short articles or conference posters, gray literature, or preprint publications.
(c) Studies whose assessments do not present the method used.
(d) Systematic review or meta-analysis studies.
(e) Studies not published in the English language.

#### Quality criteria of primary studies

To assess the quality of the study, it was analyzed whether it was published in peer-reviewed journals or annals of events when referring to articles or studies approved by the examining board of the institution when referring to undergraduate, master's, or PhD theses. To assess the articles, the following criteria were used: the population considered in the intervention and use of an eHealth strategy. Our review included eHealth intervention studies in older adults with PC. These eHealth interventions can be accessed with a computer, smartphone, or tablet.

The studies were included if the main intervention component is mediated via computer (e.g., website, web platforms, and email) or smartphone/tablet (e.g., mobile app, text messages, and phone calls). Interventions could be applied to groups or individuals, as long as they involved older adults. Interventions could involve single or repeated online contacts with the research and/or intervention teams. The modalities of intervention contacts may be educational, reminders, self-monitoring, behavioral counseling, clinical decision aid, mixed interventions, or—if not well explained—classified as “Unclear.”

#### Selection process of primary studies

Search strings were built with the keywords and their synonyms. The strings were submitted to search engines. After reading the abstract and applying the inclusion and exclusion criteria, the study was selected, and its relevance was confirmed by the first author of this article. Two reviewers independently reviewed records and all potentially relevant full-text publications. One reviewer extracted the data of 50% of the studies included, and another reviewer extracted the data for the remaining 50%; the second author of this review verified all the extracted data. One reviewer assessed the quality of the studies included, and a second one verified the assessments.

#### Information extraction strategy

**FIG. 1.**
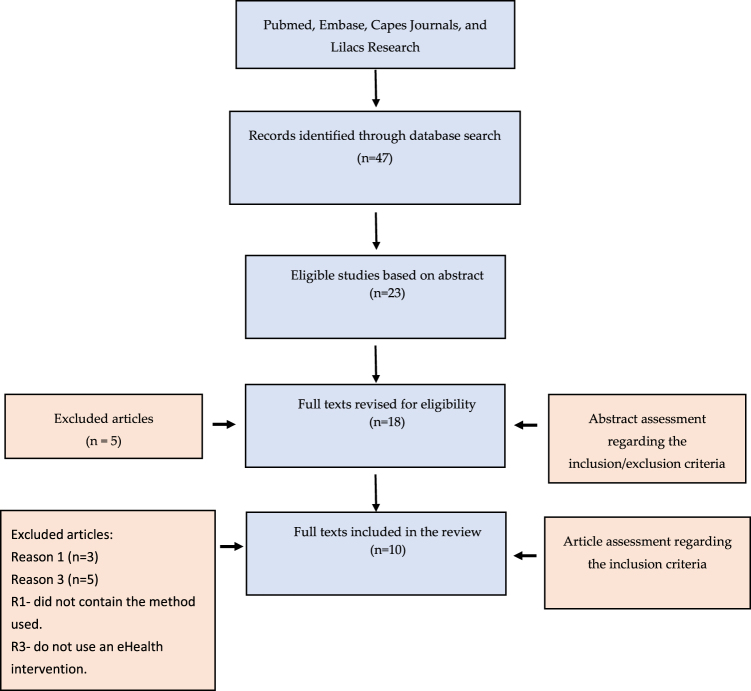
Review flow diagram.

The studies included were read in full, and the reviewer summarized each one of them, highlighting the methods used for the assessment and the parameters considered, when necessary. “Data extraction forms” were filled in for texts considered valid and read in full. In addition to basic information (bibliographic data, date published, abstract, among others), these forms contain a summary of the work. For this activity, the START software was used.^15^ The review flow diagram is detailed in Figure 1.

#### Summary and clusterization of results

The attributes that were extracted from the articles included are described in Table 2. The XLM-R-Base was used to group the articles.^16,17^ For the clustering process, “K-means” was used. K-means is an unsupervised learning algorithm, interactive, with low computational complexity,^18^ where the number of clusters (groupings) is arbitrarily assigned through a constant. Texts are labeled based on their relationship with the centroids, that is, if the text is closer to one centroid in relation to the others, it belongs to the cluster of the closest centroid and receives the label of this cluster. The elements of a cluster tend to be similar and different from those outside the group.

**Table 2.** Summary of the Studies Included in This Review (2010–2021)

ID	Author (year), journal	Study design	Study population	eHealth modality	eHealth intervention category	Brief description of the intervention	How long the intervention lasted	Conclusion
1	Song et al. (2015),^21^ *Oncology Nursing Forum*	Quantitative, qualitative, mixed-methods approach	25 Couples (1 patient with PC and 1 spouse) (62.95 years old) M (59.32) spouses	Web-based platform	Education	The intervention was web-based and focused on couples for the management of symptoms of PC, the platform is called PERC. The PERC website included seven educational modules for couples to review; two modules were mandatory and five were optional.	Each couple had a maximum of 8 weeks to complete the modules; they were asked to complete one module per week or to complete modules at their own pace.	PERC was a viable and acceptable method to reduce the side effects of symptoms related to the treatment of PC and improve the quality of life of the couples.
2	Yanez et al. (2015),^33^ *Cancer*	Randomized controlled trial	74 M (68.84 years old)	Web-based platform	Self-monitoring	The specific website for the CBSM intervention contained relaxation songs and self-guided stress management teaching tasks for the participants to review between weekly group sessions.	6 Months	Technology-based CBSM interventions can be feasible, acceptable, and effective.
3	Paterson et al. (2016),^36^ *European Journal of Oncology Nursing*	Subsample of a longitudinal study	12 M NA	Interactive smartphone application	Mixed interventions	Mobile application with an electronic behavioral diary, based on the Social Support Theory, to obtain information in real time. Self-reports were requested by an audible alarm activated 3 times a day (total of 93 times in the follow-up).	31 Days	The results demonstrated the acceptability of eHealth technology in a population of older men affected by PC. It demonstrated the need for individualization for the size of propositions based on the Social Support Theory.
4	Sundberg et al. (2017),^20^ *Supportive Care in Cancer*	Nonrandomized, controlled	130 M (69 years old)	Interactive smartphone application	Self-monitoring	Patients received a smartphone with an app installed. They were asked to submit daily reports any time they felt unwell throughout the study period.	5–8 Weeks	The study results suggest that the Interaktor app can be an effective eHealth tool to facilitate care needs during their PC treatment.
5	Golsteijn et al. (2017),^44^ *BMC Cancer*	IM	NA	Web-based platform	Mixed interventions	Intervention proposal for patients to remotely receive personalized PA counseling three times, with printed materials, and with a pedometer to set goals to improve their PA levels.	12 Months	The use of the IM protocol resulted in a systematically tailored evidence-based and theory-based intervention that provides personalized PA counseling for prostate and colorectal PC.
6	Wootten et al. (2014),^39^ *Psycho-Oncology*	Randomized controlled trial	142 M (61 years old)	Web-based platform	Behavioral counseling	MRA is a self-guided online program based on psychological intervention for the specific needs of patients with PC.	10 Weeks	The results indicate the potential of the MRA approach to provide support that men might not otherwise receive, and also highlight the importance of psychological intervention to improve sexual outcomes.
7	Clarke et al. (2019),^19^ *Supportive Care in Cancer*	Nonrandomized controlled trial	41 M (63 years old)	Web-based platform	Self-monitoring	HNA is an online self-assessment tool designed to holistically collect the needs of men who have been diagnosed with PC.	6 Months	Although the study demonstrated the feasibility of implementing the PC-sHNA approach, it did not meet the *a priori* progression criteria.
8	Tran et al. (2018),^22^ *Journal of Medical Internet Research*	Single-arm pilot trial	29 M (55 years old)	Interactive smartphone application	Mixed interventions	Strength Through Insight is a smartphone app designed to explore the feasibility and acceptability of smartphone devices as a digital health care tool to collect PROs for cancer patients in the health care environment through a mixed-methods approach.	12 Weeks	Strength Through Insight has been demonstrated as an achievable and acceptable data collection method for ePROs. Almost all respondents reported that using the smartphone app is easier or equivalent to the traditional pen-and-paper approach, providing evidence of acceptability and support for using remote PRO monitoring.
9	Evans et al. (2020),^45^ *International Journal of Behavioral Medicine*	Qualitative study	18 M (71.5 years old)	Web-based platform	Education	A semi-structured interview that explored the participants' experiences and their understanding of the disease, their physical activity levels, the advice received from HCPs, as well as their acceptability and suggested content for an eHealth tool.	43 Min	The results identified key aspects that were useful for the design of person-centered exercise programs. The participants were positive about the web-based tool and expressed the need for individualized, easy-to-use, and reliable information with the support of an integrated professional.
10	Evans et al. (2021),^46^ *Pilot and Feasibility Studies*	Randomized controlled trial	66 M	Web- and telephone-based platform	Mixed interventions	Exercise guide	8 Weeks	The study aims to determine the potential feasibility of an online, remotely monitored exercise intervention developed for individuals with metastatic PC. If feasible, this pilot intervention will inform the design and implementation of other distance-based interventions.

CBSM, cognitive behavioral stress management; HCPs, health care professionals; HNA, holistic needs assessment system; IM, intervention mapping; M, men; MRA, My Road Ahead; NA, not applicable; PA, physical activity; PC, prostate cancer; PERC, Prostate Cancer Education and Resources for Couples; sHNA, specific holistic needs assessment.

**FIG. 2.**
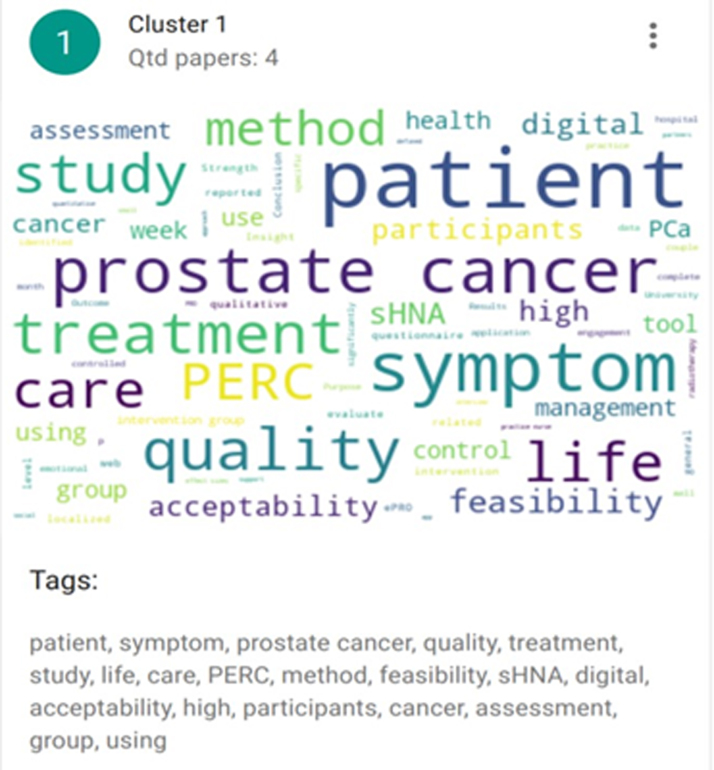
Grouping of Cluster 1 articles. Articles included in this cluster (ID articles): 1, 7, 4, and 8.

**FIG. 3.**
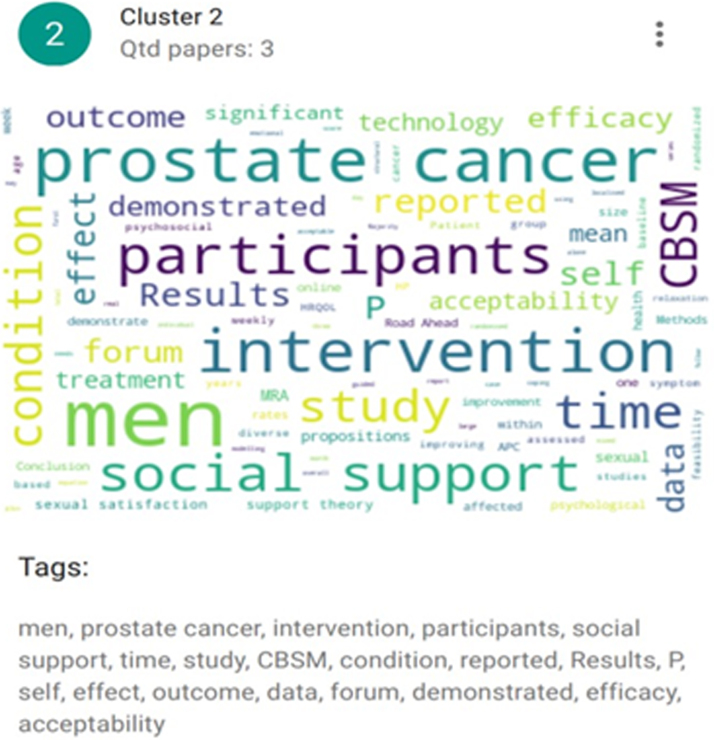
Grouping of Cluster 2 articles. Articles included in this cluster (ID articles): 2, 3, and 6.

**FIG. 4.**
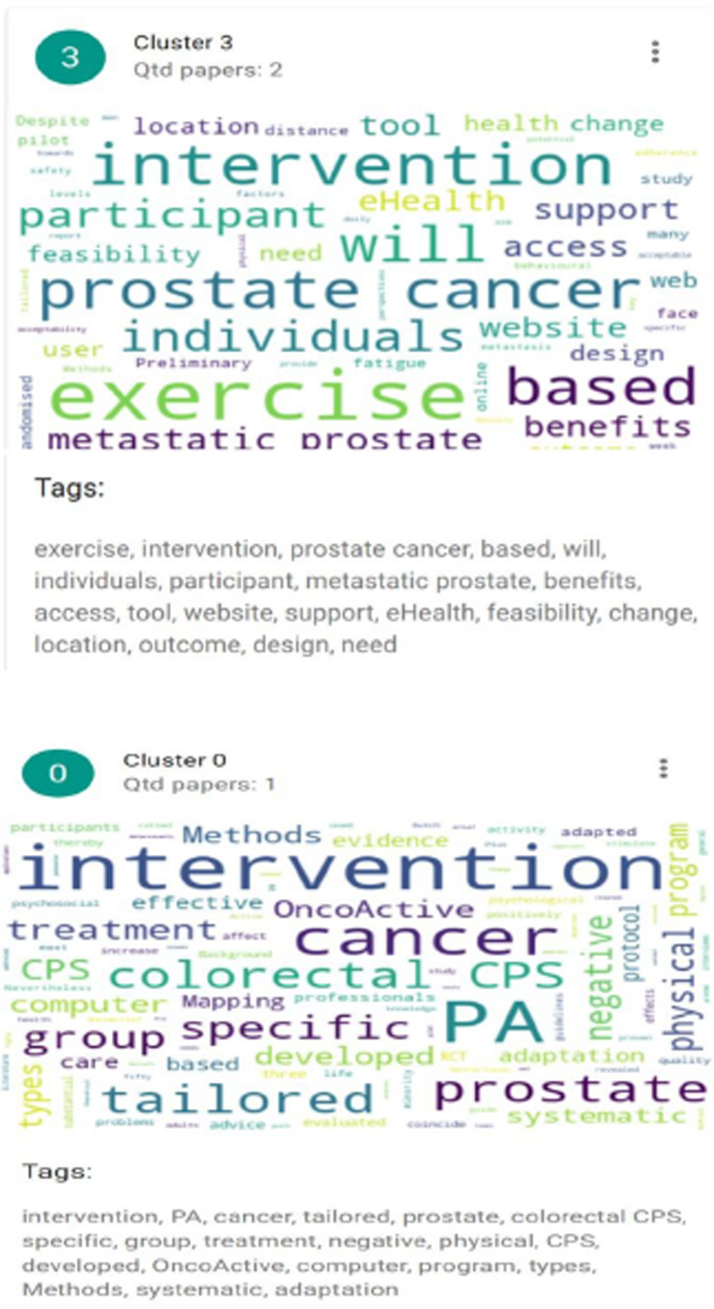
Grouping of Clusters 3 and 0 articles. Articles included in these clusters (ID articles): 5, 9, and 10.

The K-means algorithm runs until there are no more element movements between clusters or until the maximum number of interactions has been reached (Figs. 2–4). Clustering was used to present the results and discuss the articles included. And they were defined according to their themes: Cluster 1 (eHealth interventions: integrated care, detection of symptoms, and quality of life in older patients with PC—ID articles 1, 4, 7, and 8), Cluster 2 (Psychological eHealth interventions in older adults with PC—ID articles 2, 3, and 6), and Clusters 3 and 0 (Physical activity eHealth interventions in older adults with PC—ID articles 5, 9 and 10).

## Results and Discussion

### Summary of study characteristics

Initially, a total of 47 articles were found in the selected databases: 13 (28%) in PubMed, 6 (13%) in Embase, 17 (36%) in Capes Journals, and 11 (23%) in Lilacs-BVS (Table 1). Of these, 24 articles were duplicated, leaving 23 articles to be analyzed according to the inclusion and exclusion criteria. After this analysis, five articles were excluded. Using the Cochrane review protocol, 18 articles were reviewed for eligibility. Eight were excluded, and therefore, 10 articles were included in the review (Fig. 1).

Among the included studies, an alternative pattern was identified in the number of articles published per year; even though the search for the review was carried out between 2010 and 2021, only articles in the period between 2015 and 2021 were included in this study. Only the *International Journal of Behavioral Medicine* was cited more than once in this review (*n* = 2). Randomized controlled trial (RCT) (*n* = 3) and nonrandomized controlled trial (*n* = 2) study designs were the most frequent. Thus, study designs were heterogeneous, with method mixing, subsampling of a longitudinal study, intervention mapping (IM), single-arm pilot trial, and qualitative study.

For a better presentation and discussion of the results, we performed the classification of modalities and categories of eHealth interventions. Two types of modalities were identified and classified in the articles of this review, the interventions that used a web-based platform (WBP) were the most common (*n* = 7), followed by interactive smartphone application (ISA) (*n* = 4). The article by Evans et al. was included in both modalities, as it uses both WBP and ISA. As for the classification, mixed interventions were the most used (*n* = 4), followed by self-monitoring (*n* = 3), educational (*n* = 2), and behavioral counseling (*n* = 1). The time of interventions ranged from 31 days to 12 months.

The study by Evans et al. was carried out in a sectional way and the interview lasted an average of 43 min. All studies assessed the acceptance, feasibility, and efficiency of using eHealth by patients and/or health care professionals (HCPs).

### eHealth interventions: models of integrated care, symptom detection, and quality of life in older patients with PC

Integrated care models have been suggested as a way to optimize cancer treatment and follow-up. Among these models, the holistic needs assessment (HNA) aims to provide a mechanism to help patients identify and more easily disclose their needs, as well as assist HCPs in developing a people-centered care approach. In our review, we identified a study that developed a specific online holistic approach to assessing the needs of patients with PC—the PC-specific holistic needs assessment (sHNA).^19^ The sHNA has proven to be useful in identifying “red flag” symptoms (e.g., threatening their physical or psychological integrity), which, when identified in real time by the HCP, prompts the patient to urgently contact their general practitioner.

Thus, it contributes to the nurses' decision-making on when the patient should seek further medical assistance. There was also a high level of acceptability by patients and HCPs. However, viability results of the sHNA did not meet the progression criteria for conducting a randomized clinical trial in the primary care setting. The lack of integration between the IT platform and the National Health Services Clinical Trust data management system was the main limiting factor. Therefore, further research into eHealth using the sHNA model may facilitate adequate communication between patients and HCPs. Furthermore, even with the benefits described in the literature of this care model, it is rarely performed in clinical practice.

Older patients with PC undergoing radiotherapy treatment suffer a variety of symptoms, such as bladder and bowel malfunction, pain, anxiety, distress, and sleep issues that affect their HRQOL. Therefore, eHealth interventions for the early detection and management of symptoms related to the treatment of PC are useful. In this sense, our review included a study that assessed Interaktor, a mobile or tablet application.^20^ This application enables patients to assess symptoms in real time and sends alert messages when they do not submit their symptom assessment reports. At the clinic, the health team, based on these reports, performs the risk analysis, prepares the patient-centered care approach, and conducts the procedure.

The app demonstrated a positive effect on early detection of symptoms and HRQOL. Therefore, improving management and communication between patient and HCPs, especially in real time through eHealth, is a field of research and clinical practice to be explored. Conducting larger RCT studies to assess clinical efficacy, feasibility, usability, and acceptability of users and HCPs before the general implementation of ISAs is a challenge. However, for Ritterband et al.,^8^ carrying out interventions with RCT study designs enables researchers to have large sample sizes (not being geographically limited), thus enabling better mediating and moderating treatment tests.

In eHealth interventions, few studies include both patients and their spouses in their designs. A study included in our review assessed an educational WBP called Prostate Cancer Education and Resources for Couples (PERC) to support patients and their spouses.^21^ The PERC platform helps couples work together through tasks that are organized to alleviate the impact of the patients' symptoms after PC treatment. It is designed for older adults, a population that has a relatively large proportion of people with little experience in using computers and the internet—even though high usage rates were found. The participants “as easy to use and understand,” and assessed it as “engaging, high quality, and relevant.”

Therefore, this eHealth model can be useful for patients who live far from cancer treatment centers and, since it is an educational modality, it can improve our low health literacy rates, in addition to the integration of couples that this model provides.

eHealth tools to collect the electronic patient-reported outcomes (ePROs) of patients with PC have been shown to increase survival rates and improve their HRQOL.^22^ However, for ePROs to be routinely integrated into clinical practice, data collection methods must be efficient, proving to be instantaneous, inexpensive, reliable, and clinically feasible. In this sense, we included a study that assessed the feasibility and acceptability testing of an ISA, called Strength Through Insight, developed on Apple's ResearchKit platform to electronically collect ePROs from patients with PC. For testing the application, the quality of life and Expanded Prostate Cancer Index Composite (EPIC-26)^23^ instruments were used, and patients with advanced PC answered the EPIC-CP and FAPSI-8 instruments.^24^

Additionally, questions were asked for the qualitative assessment of the application. The study results confirmed that Strength Through Insight is an achievable and acceptable data collection method for ePROs. In this direction, the researchers have found that ePROs can serve as an innovative way to increase patient involvement in self-care, for the management of reported symptoms and clinical decision-making, thus facilitating meaningful interactions with the provider for the development of a personalized treatment.

Quality of life in patients with PC was an outcome found in the articles included. Specific HRQOL instruments for patients with PC were used, such as the EuroQol five dimensions questionnaire (EQ-5D),^25^ European Organization for Research and Treatment of Cancer Quality of Life Questionnaire (EORTC-QLQ), versions: QLQ-C30 and QLQ-PR25,^26,27^ Functional Assessment Scale of Chronic Illness Therapy–General (FACT-G),^28^ and EPIC-26.^23^ eHealth interventions have been shown to improve the HRQOL of patients with PC. This is an interesting outcome to be analyzed for assessing these interventions. For Di Maio et al.,^29^ the HRQOL is essential to assess the results of eHealth clinical trials.

### Psychological eHealth interventions in older adults with PC

Psychological suffering was reported by 38% of patients with PC; however, the care needs of these sufferings are not met.^30^ As an example, a study by Smith et al.^31^ identified that 54% of patients with PC expressed some level of unmet psychological need. In this sense, evidence-based interventions to maximize quality of life and psychological adjustment are needed for these men. Studies that analyzed technology-based psychosocial interventions to reduce symptom burden, better self-care management in their health care, and improved HRQOL in men with PC have shown to be achievable, effective, and with good acceptability.^20,32^

Among the psychosocial interventions, studies that used the cognitive behavioral stress management (CBSM) model for men with PC showed improving emotional levels, well-being, HRQOL, and sexual functioning.^33,34^ However, testing the CBSM in patients with advanced-stage PC is one of the gaps in the literature. In this sense, we included a study that verified that the CBSM intervention in this patient profile, through a WBP. The results showed that there was an improvement with clinical significance, the symptoms of depression, self-efficacy, distress, and functional well-being of patients, even after 6 months of intervention.

Another psychosocial intervention strategy is the ecological momentary assessment (EMA), which is a strategy able to assess patients' self-reports about their symptoms in real time, through an ISA. This type of assessment facilitates real-time data collection, reduces data production concerns, and reduces retrospective memory recall bias. This helps to identify gaps in the patient's self-management experiences to develop personalized supportive care interventions. However, further studies are needed to incorporate and test new eHealth strategy designs in patients with PC.^35^

In our review, we included a study that assessed the effect of an intervention in the EMA model, based on the “Theory of Social Support”^36^ with ISA for older adults at different stages of PC. The evidence found in this study reinforces the need to understand, in real time, which strategies patients are using to face and reduce their symptoms; it is worth noting that each patient experiences a variety of symptoms. Therefore, health professionals can use this eHealth model to support and adapt self-care to individualize the patient-centered care plan in a timely manner.

Psychosexual problems are recurrent in patients being treated for PC. Erectile dysfunction and urinary incontinence are common side effects. Previous studies have shown that psychological interventions that used the online model in patients with localized PC were well accepted, especially in psychosexual issues, due to the guarantee of relative anonymity and easy access to information and support.^37–39^ However, many men still report having difficulty in accessing adequate care.^39^ In this sense, we included in our review an RCT study that found a significant positive effect of the eHealth intervention called “My Road Ahead (MRA)” on the total sexual satisfaction of patients with localized PC.

The gain in sexual satisfaction had clinical implications, with greater effect when the patient has access to both the WBP (WBP-MRA) and a forum for discussion among peers. Interestingly, this improvement in sexual functioning was not associated with the use of aids for erectile dysfunction, quite the other way around, there was a decrease in the use of these aids throughout the study, which can be explained as the Forum is an opportunity to discuss, and possibly normalize, their experience with peers while at the same time learning new skills. Additionally, other conditions such as time of diagnosis and treatment, stage of PC, and psychosocial conditions must be analyzed to understand the effect of this “enhanced” approach (MRA + Forum).

### Physical activity eHealth interventions in older adults with PC

The physical, psychological, and social problems that affect older adults with PC promote a decrease in the level of physical activity. Researchers highlight that only 30–47% of patients with PC meet their physical activity guidelines.^40^ However, previous studies demonstrated the beneficial effects of physical activity in patients with PC, including symptom management, rehabilitation, and long-term survival rates.^41–43^ In this line, Hart et al.^41^ discussed the possible hypotheses of the physiological mechanisms of these benefits, for example, it improves immune function, modulates circulating factors (such as insulin and growth factors), reduces inflammation, and improves the effectiveness of the treatment.

In this sense, eHealth studies have shown to increase the level of physical activity in patients with both localized and metastatic PC, as a consequence, it improves HRQOL and cancer-related symptoms.^44–46^

Among the different exercise protocols that are used in clinical routine, the home-based approach is an alternative with high adherence and participation levels in patients with PC.^47^ After using the inclusion and exclusion criteria, we included the study by Golsteijn et al.,^44^ which described the process of systematic development and analyzed an eHealth intervention called OncoActive to stimulate regular physical activity and leisure of these patients.

This tool was developed using IM, a type of methodology to systematically adapt an intervention^48^—in this case, Active Plus.^49,50^ In this study, the use of the IM approach proved that the content produced from the OncoActive intervention is considered adequate to the needs and preferences of the target group, and this provides personalized physical activity counseling to patients. Continuing the OncoActive studies, Golsteijn et al.^44^ found the positive effect of the tool in improving the levels of self-reported physical activity, physical function, fatigue, and depression, especially after 6 months of intervention.

eHealth interventions to identify and alter physical activity levels in patients with metastatic-type PC is an underexplored field of research. We included two studies that described eHealth strategies for these patients. The first was a qualitative study that used a WBP to collect and analyze the perspectives, capacity, and needs of end users in the development of an eHealth tool for specific exercises for patients with PC. Regarding physical activity, some obstacles were identified, such as their financial condition, individuals who disliked going to the gym, boredom, and motivation after diagnosis. However, facilitations such as social and medical support and understanding the benefits of physical activities were described.

In addition, all patients had positive attitudes toward eHealth interventions. Continuing the first study, a mixed type “Study Protocol” (WBP + ISA) was carried out to analyze the feasibility of remotely monitored exercises in patients with metastatic PC, called “Exercise Guide”.^46^ The exercise protocol used was the one proposed by Galvão et al.^51^ plus the isometric training of the paravertebral muscles proposed by Rief et al.^52^ The proposal also included a telehealth coaching to monitor progress throughout the intervention, for a previous study showed that the inclusion of human support increases the effectiveness of online interventions.^53^

Some barriers were found regarding the implementation and quality assessment of this tool, for example, patients living in remote areas with poor internet access, adherence to behavioral change and physical activity interventions, and difficulty in recruiting older men for health-related interventions. According to the Exercise Guide website (https://www.exerciseguide.org.au/eng/pages/homepage), this group of researchers is continuing the studies to verify the effect of the eHealth tool, and the results are in the final analysis stage and will be fully reported.

### Study strengths and limitations

There are some limitations in our review. First, only articles in English, published in peer-reviewed journals, were reviewed. Second, the study did not include any contact with oncology experts, so some relevant studies may not have been included. Third, a formal quality assessment of the included studies was not conducted. This would have provided more detail on some of the methodological strengths and limitations of the studies. This area (eHealth interventions for patients with PC) is still a little explored field. Although some variability was found in intervention methodologies, we do not ensure the greatest methodological rigor, including only a RCT or a controlled clinical trial.

Some interventions were clearly in the experimental phase, whereas others were further ahead in development and testing. Future research into such interventions should consider intervention fidelity and the implementation process. Even though this was a quick review of evidence, a rigorous review approach was maintained through: (1) the analysis of relevant systematic references and the assessment of narrative reviews for the inclusion of documents; (2) titles and abstracts were reviewed by two team members; (3) full-text inclusion was conducted independently by two people with experience in conducting high-quality systematic reviews, with the included studies being reviewed by a third person who was also a member of the synthesis team; and (4) a team member completed all data extraction, and this was verified by a second person.

## Conclusions

This quick review aimed to identify and analyze eHealth intervention programs for older adults with PC. These interventions for PC patients are relatively new but promising in supporting the care options that are currently offered. The findings in this quick review suggest that web-based (eHealth), WBP, ISA, or mixed interventions can result in improved real-time symptom detection, total sexual satisfaction, emotional level, well-being, self-management, level of physical activity, general health, and HRQOL. The range of outcomes identified in the studies can also be explained by the diversity of eHealth categories used, such as educational, self-monitoring, behavioral counseling, and mixed.

It was not the aim of this review to compare which types of web-based interventions are most effective and the reasons for our conclusion. Important potential benefits of eHealth interventions may be less expensive than those that exclusively involve face-to-face support from professionals, thus being more accessible to patients and HCPs. The HRQOL outcome was used in most studies and proved to be an important analysis variable to verify the effects of eHealth tools in these patients.

We recommend monitoring the studies that are currently in the experimental phase, study protocol, or IM for future assessments of the effect and implementation of eHealth tools in clinical practice. We suggest further research with eHealth interventions in patients with PC at different stages of the disease, from socioeconomic levels, at different stages of diagnosis and treatment, and HRQOL.

## Abbreviations Used

APC: advanced-stage prostate cancer
CBSM: cognitive behavioral stress management
EMA: ecological momentary assessment
EORTC-QLQ: European Organization for Research and Treatment of Cancer Quality of Life Questionnaire
EPIC-26: Expanded Prostate Cancer Index Composite
ePRO: electronic patient-reported outcome
EQ-5D: EuroQol five dimensions questionnaire
FACT-G: Functional Assessment Scale of Chronic Illness Therapy–General
HCP: health care professional
HNA: holistic needs assessment
HRQOL: health-related quality of life
IM: intervention mapping
ISA: interactive smartphone application
MRA: My Road Ahead
NA: not applicable
PA: physical activity
PC: prostate cancer
PERC: Prostate Cancer Education and Resources for Couples
PRO: patient-reported outcome
RCT: randomized controlled trial
sHNA: specific holistic needs assessment
WBP: web-based platform
